# Practical Electricity in Medicine and Surgery

**Published:** 1890-09

**Authors:** 


PRACTICAL ELECTRICITY IN MEDICINE AND SURGERY. By G. A. Liebig, Jr., Ph. D., and George H. Rohé, M. D.

Philadelphia: 1231 Filbert Street. F. A. Davis, publisher. 1890.

This is the latest work upon electro-therapeutics. It is divided into three parts: Part I, a systematic description of electricity, magnetism, and batteries. Part II is devoted to electro-physiology, electro-diagnosis, and electro-medical apparatus. Part III gives the therapeutic effects of electricity and methods of application, with illustrations of curative results, especially in a case of elephantiasis arabum.

It is, of course, all empirical, but very instructive and stimulating in its tendency upon the reader, stimulating him to the desire to investigate further and make discoveries for himself. We cannot say much in praise of the description given of practical electrical units—ohms, volts, etc.

For the make-up of the book we can say it is well printed, profusely illustrated, and handsomely bound. W. M. J.

